# Fetal Cardiac Function in Early Labour and Intrapartum Outcomes: A Prospective Observational Study

**DOI:** 10.1111/1471-0528.18224

**Published:** 2025-05-21

**Authors:** Andrea Dall'Asta, Chiara Melito, Beatrice Valentini, Mariagrazia Capurso, Maria Teresa Baffa, Olga Patey, Basky Thilaganathan, Tullio Ghi

**Affiliations:** ^1^ Department of Medicine and Surgery, Obstetrics and Gynecology Unit University of Parma Parma Italy; ^2^ Fetal Medicine Unit, St George's University Hospitals NHS Foundation Trust University of London London UK; ^3^ Royal Brompton and Harefield Hospitals, Guy's & St Thomas' NHS Foundation Trust London UK; ^4^ Molecular and Clinical Sciences Research Institute, Vascular Biology Research Center St George's University of London London UK; ^5^ Department of Women and Child Health, Catholic University of Sacred Heart Fondazione Policlinico Universitario Agostino Gemelli IRCCS Rome Italy

**Keywords:** fetal cardiac function, intrapartum fetal distress, labour, obstetric interventions, placental insufficiency

## Abstract

**Objective:**

To assess fetal myocardial deformation in normo‐oxygenated foetuses in early labour and its relationship with intrapartum outcomes.

**Design:**

Single centre prospective study.

**Setting:**

Referral tertiary maternity unit.

**Population:**

Uncomplicated singleton term pregnancies in early labour.

**Methods:**

Two‐dimensional (2D) ultrasound clips of the 4‐chamber view of the fetal heart were collected in labour and sent to TomTec software for the offline speckle tracking echocardiography analysis. The left (LV) and right ventricular (RV) myocardial (MyoGLS) and endocardial longitudinal (EndoGLS) strain were evaluated.

**Main Outcome Measures:**

Operative delivery including caesarean or assisted vaginal birth due to suspected intrapartum fetal compromise (IFC) as defined by standard CTG criteria.

**Results:**

In total, 208 cases were included. Operative delivery due to suspected IFC was recorded in 20 (9.6%) cases and was associated with higher LV ejection fraction (EF) (47.4 + 8.2 vs. 40.9 + 12.9%, *p* = 0.03) and increased RV MyoGLS (−15.9 + 4.0 vs. −12.5 + 4.3%, *p* < 0.01) and RV EndoGLS (−17.7 + 4.4 vs. −14.3 + 4.7%, *p* < 0.01) compared to cases not having operative delivery due to suspected IFC. Maternal age (OR 1.138, 95% CI [1.010–1.281], *p* = 0.03), baseline fetal heart rate at acquisition (OR 1.068, 95% CI [1.007–1.134], *p* = 0.03) and RV MyoGLS (OR 0.575, 95% CI [0.366–0.903], *p* = 0.02) were independently associated with the primary outcome.

**Conclusions:**

Increased right ventricular myocardial deformation is associated with operative delivery due to suspected IFC, suggesting an early cardiac response to labour‐related hypoxia.

## 1 Introduction

Labour induces intermittent hypoxia due to uterine contractions, necessitating fetal cardiovascular adaptation. Indeed, uterine contractions reduce uteroplacental perfusion by 60% [1] and lead to a reduction of the fetal PaO2 by approximately 25% with each contraction [2]. Although most foetuses can cope with such intermittent episodes of hypoxemia during labour, in some foetuses, sustained hypoxia and acidemia may develop, leading to intrapartum injury and adverse outcomes such as death or cerebral palsy [3, 4, 5, 6, 7]. The fetal heart is a key organ in the process of adaptation to intrauterine hypoxia [8, 9]. The fetal adaptive cardiovascular responses to the hypoxic stress are sustained by both parasympathetic and sympathetic activities [8, 9].

The antenatal assessment of fetal myocardial deformation by speckle tracking echocardiography (STE) is considered a sensitive tool to assess cardiac function and to investigate the earliest hypoxic‐related adaptive changes in the fetus [10]. STE has been widely used in paediatric cardiology [11, 12] and, more recently, investigated for monitoring fetal wellbeing, particularly in pregnancies complicated by placental insufficiency [10, 13]. The aim of this study was to assess myocardial deformation in a cohort of normo‐oxygenated foetuses during the early phase of labour and to investigate its relationship with labour outcome.

## 2 Methods

The study was conducted following the STROBE Guidelines [14] and approved by the Ethics Committee of the University Hospital of Parma [270/2018/OSS/AOUPR]. Informed consent was obtained from each participant prior to enrolment. This was a single centre prospective study including a non‐consecutive cohort of women with singleton term pregnancies delivering at a tertiary maternity unit between April 2022 and April 2024. The inclusion criteria for the study were: uncomplicated singleton pregnancy in active phase of labour, gestational age at enrolment between 37^+0^ and 41^+3^ weeks, spontaneous onset of labour, normal estimated fetal weight at third trimester ultrasound, no evidence of fetal hypoxia based on a normal cardiotocography (CTG) upon labour admission as defined according to the FIGO 2015 criteria [15]. Cases were excluded from enrolment in the event of any prenatally suspected or postnatally confirmed structural or genetic anomaly, conditions potentially impacting maternal and fetal haemodynamic such as preeclampsia, chorioamnionitis as defined according to the Gibbs criteria [16] at recruitment, and inability to obtain a two‐dimensional ultrasound clip of the 4‐chamber view allowing the clear visualisation of the endomyocardial borders.

Demographic and clinical characteristics were recorded and included maternal age and ethnicity, parity, gestational age at the onset of labour, BMI at booking and at delivery, intrapartum features such as the baseline FHR, the administration of oxytocin, and the perinatal outcomes including mode of delivery and birthweight [17]. The primary outcome of the study was the accomplishment of operative delivery including caesarean section or assisted vaginal birth due to suspected IFC. This was defined subjectively by the physician in charge of the patient care based on abnormal CTG tracing, according to the FIGO classification system [15].

In the eligible cases, 2D ultrasound (US) clips of the 4‐chamber view of the fetal heart were collected in the early stage of labour, defined as a cervical dilatation ≥ 4 cm in the presence of at least 3 contractions every 10 min. All the US acquisitions were performed by members of the research team with dedicated expertise in prenatal US using a portable machine Samsung HM70EVO equipped with a 5‐MHz convex transducer. The clips were acquired during fetal rest, with the pregnant woman holding her breath for a few seconds to avoid artefacts and in between uterine contractions. The 2D images were acquired with a high (i.e., ≥ 80 Hz) frame rate (FR) achieved by optimisation of the gain, depth and sector width. To perform the off‐line myocardial strain analysis, a minimum of 3 cardiac cycles were recorded. The clips were digitally stored in a standard Digital Imaging and Communications in Medicine (DICOM) format.

**FIGURE 1:**
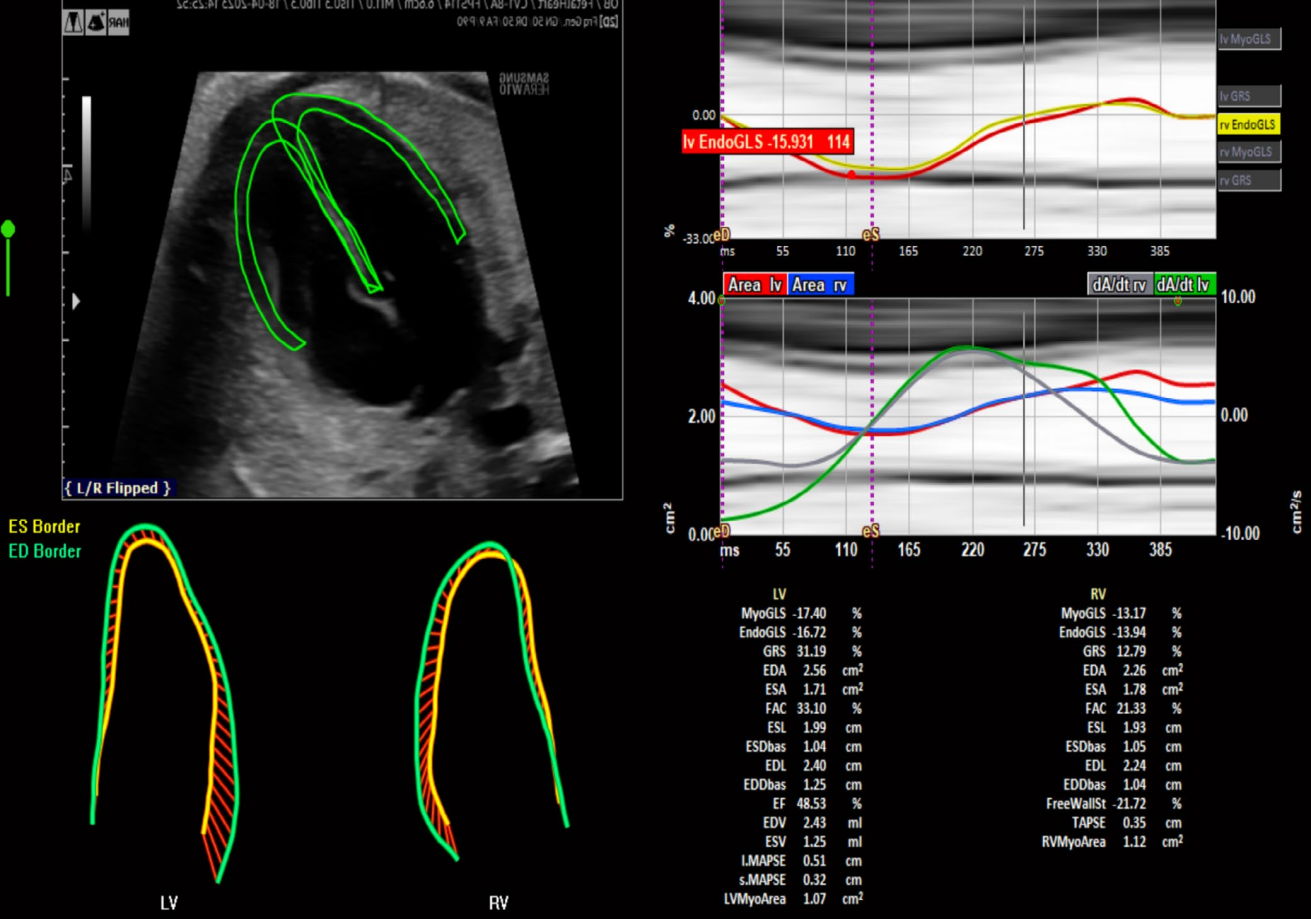
Interface of the TomTec software. Left: 4‐chamber view showing the contours of myocardium of both ventricles tracked. Right: data output for each ventricle includes the indicators of cardiac morphometry and function including deformation (i.e., cardiac strain).

The stored DICOM data were examined offline by one single member of the research group (CM) with expertise in STE analysis using the fetal version of TomTec GmbH (Munich, Germany). STE analysis using TomTec GmbH is semi‐automated, and previous data support its intra‐ and inter‐operator reproducibility [18, 19, 20]. Briefly, a prerequisite for offline analysis is the identification of the beginning and the end of each cardiac cycle by M‐Mode. Then, the researcher is required to mark the endocardial border with three dots: one on the junction of the septal wall annulus, one on the junction of the lateral wall annulus, and the last on the apex of the ventricle. Automatically, the endocardial border of the ventricle is traced with an inner line and an outer line delimitating the epicardium. Manual adjustments can be performed when deemed necessary to optimise the delimitation of the contour of the cardiac walls. Clips of the left and the right ventricles were analysed simultaneously by one single member of the research team (CM). All the morphometric and functional parameters of interest were automatically computed by the TomTec GmbH software (Figure 1). However, for the purposes of this study, only the functional indicators of cardiac deformation (i.e., cardiac strain) were considered.

Statistical analysis was performed using IBM SPSS Statistics for Windows, Version 25.0 (SPSS Inc., Chicago, IL, United States). Descriptive statistics were presented as follows: continuous variables were described by the mean and standard deviation (SD), and categorical variables were expressed by absolute number or percentage. Normal vs. abnormal distribution was evaluated by means of the Kolmogorov–Smirnov and Shapiro–Wilk tests. Student's *t*‐test and Mann–Whitney *U* test were used to compare continuous variables as appropriate, while the chi‐square test was used in the analysis of proportions between the cases having versus those not having an operative delivery due to suspected IFC. *p* < 0.05 was considered statistically significant for all comparisons. The study was performed following the STROBE guidelines [21].

## 3 Results

**FIGURE 2:**
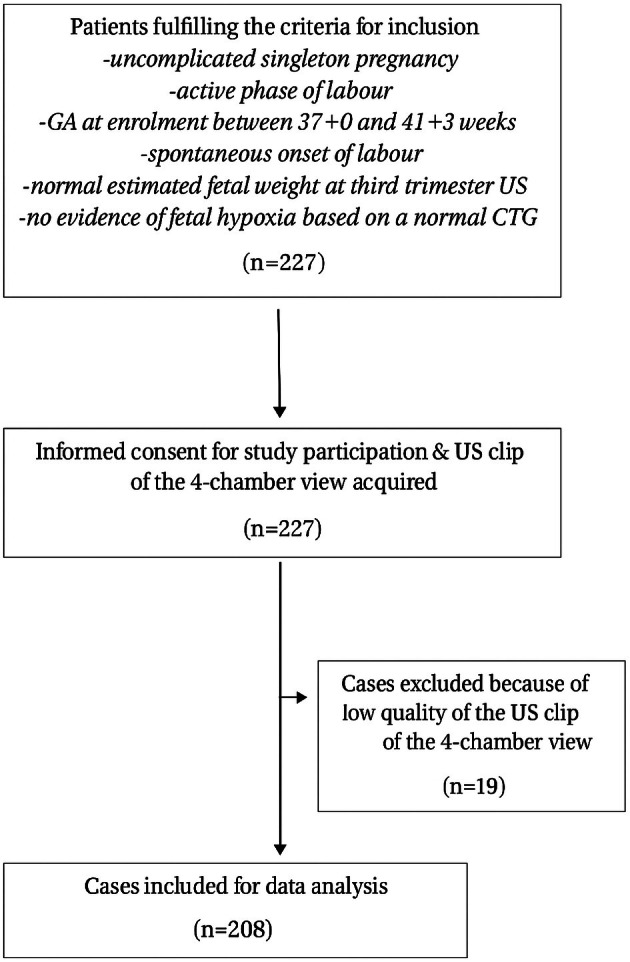
Flow chart (according to STROBE guidelines) for inclusion of cases. CTG, cardiotocography; GA, gestational age; US, ultrasound.

Overall, 227 women fulfilled the inclusion criteria and gave informed consent for study participation; 19 cases were excluded from data analysis because of the low quality of the 4‐chamber view US clips, leaving 208 cases for data analysis (Figure 2). Operative delivery due to suspected IFC was recorded in 20 (9.6%) cases and was accounted for by 17 instrumental deliveries and 3 caesarean births. The mean birthweight was 3364 ± 413 g with 16 neonates recorded as SGA (7.7%). The demographic and obstetric features of the study population in relation to the occurrence of the primary outcome are shown in Table 1.

**TABLE 1:** Demographic, intrapartum and postnatal features of the included cases in relation to the occurrence of obstetric intervention (OI) due to suspected intrapartum fetal compromise (IFC).

	OI due to suspected IFC *N* 20	Non OI due to suspected IFC *N* 188	*p*
Maternal age, years Mean ± SD	34.3 ± 5.0	31.8 ± 5.3	0.04
Ethnicity *N* (%)	Caucasian 17 (85%) African 1 (5%) Asian 0 (0%) Other 2 (10%)	Caucasian 145 (77.1%) African 22 (11.7) Asian 12 (6.4%) Other 9 (4.8%)	0.38
Nulliparity *N* (%)	17 (85%)	120 (63.8%)	0.06
Previous caesarean delivery *N* (%)	2 (10%)	14 (7.4%)	0.68
Booking BMI, kg/m2 Mean ± SD	24.2 ± 4.2	23.6 ± 4.8	0.60
IVF conception *N* (%)	2 (10%)	10 (5.3%)	0.39
Smoking *N* (%)	3 (15%)	14 (7.4%)	0.24
GBS colonisation *N* (%)	2 (10%)	34 (18.1%)	0.36
Gestational age at recruitment, weeks^+days^ Mean ± SD	40^+1^ ± 0^+6^	39^+6^ ± 1^+1^	0.22
Baseline FHR at recruitment, bpm Mean ± SD	146 ± 3	141 ± 10	0.02
Number of contractions at recruitment Median (range)	4 (3–5)	4 (3–5)	0.21
Oxytocin administration *N* (%)	14 (70%)	123 (65.4%)	0.68
Epidural *N* (%)	19 (95%)	162 (86.2%)	0.26
Meconium stained amniotic fluid *N* (%)	2 (10%)	28 (14.9%)	0.55
Duration of ruptured membranes, minutes Mean ± SD	710 ± 369	792 ± 1109	0.75
First stage length, minutes Mean ± SD	460 ± 263	334 ± 199	0.02
Second stage length, minutes Mean ± SD	87 ± 46	54 ± 52	0.01
Birthweight, grams Mean ± SD	3340 ± 363	3366 ± 418	0.79
Birthweight percentile Mean ± SD	47.4 ± 28.4	51.3 ± 28.7	0.56
Small for gestational age *N* (%)	3 (15%)	13 (6.9%)	0.20
Arterial pH Mean ± SD	7.06 ± 0.10	7.11 ± 0.13	0.12
Apgar at 5 min Median (range)	10 (5–10)	10 (2–10)	0.21

Abbreviations: BMI, body mass index; FHR, fetal heart rate; GBS, group B streptococcus; IVF, in vitro fertilisation.

The mean maternal age was higher in cases submitted to operative delivery due to suspected IFC (34.3 ± 5.0 vs. 31.8 ± 5.3 years, *p* = 0.04), in such cases, a higher baseline FHR was recorded (146 ± 3 vs. 141 ± 10 beats per minute, *p* = 0.02) and the mean duration of the first and second stage of labour was longer (460 ± 263 vs. 334 ± 199 min, *p* = 0.02 and 87 ± 46 vs. 54 ± 52 min, *p* = 0.01, respectively).

The results of the STE analysis in foetuses delivered by operative delivery due to suspected IFC compared to those who did not are shown in Table 2. Cases where operative delivery due to suspected IFC was performed showed higher LV EF (47.4 ± 8.2 vs. 40.9 ± 12.9%, *p* = 0.03) and increased RV MyoGLS (15.9 ± 4.0 vs. −12.5 ± 4.3%, *p* < 0.01) and RV EndoGLS (−17.7 ± 4.4 vs. −14.3 ± 4.7%, *p* < 0.01) compared to those not having operative delivery due to suspected IFC. No differences were noted with respect to the indicators of cardiac morphometry and function measured using STE as well as with the sphericity index of the left and right ventricles in relation to the primary outcome (Table S1). At logistic regression analysis, maternal age (OR 1.138, 95% CI [1.010–1.281], *p* = 0.03), baseline fetal heart rate at acquisition (OR 1.068, 95% CI [1.007–1.134], *p* = 0.03) and right ventricular MyoGLS (OR 0.575, 95% CI [0.366–0.903], *p* = 0.02) proved to be independently associated with the primary outcome.

**TABLE 2:** Speckle tracking echocardiography analysis of the indicators of cardiac deformation in relation to the occurrence of obstetric operative delivery (OD) due to suspected intrapartum fetal compromise (IFC).

	OD due to suspected IFC *N* 20	Non OD due to suspected IFC *N* 188	*p*
LV MyoGLS, % Mean ± SD	−17.5 ± 3.7	−15.3 ± 4.9	0.05
LV EndoGLS, % Mean ± SD	−19.4 ± 4.2	−17.3 ± 5.0	0.07
LV FAC, % Mean ± SD	32.6 ± 7.2	30.1 ± 9.4	0.24
LV EF, % Mean ± SD	47.4 ± 8.2	40.9 ± 12.9	0.03
RV MyoGLS, % Mean ± SD	−15.9 ± 4.0	−12.5 ± 4.3	< 0.01
RV EndoGLS, % Mean ± SD	−17.7 ± 4.4	−14.3 ± 4.7	< 0.01
RV FAC, % Mean ± SD	26.2 ± 7.4	24.9 ± 8.4	0.48

Abbreviations: EF, ejection fraction; FAC, fractional area change; GLS, global longitudinal strain; GRS, global radial strain; LV, left ventricle; RV, right ventricle.

Table 3 summarises the comparison of the demographic and obstetric features and the STE indices of cardiac deformation between SGA and non‐SGA neonates. Differences in ethnicity were observed (*p* < 0.01). Furthermore, the postnatal confirmation of SGA was associated with a lower frequency of epidural (68.8% vs. 88.5%, *p* = 0.02) and a shorter duration of the first stage of labour (202 ± 111 vs. 357 ± 209 min, *p* < 0.01), with no difference in indices of myocardial deformation observed in relation to the size of the neonate at birth.

**TABLE 3:** Demographic, intrapartum and postnatal features and speckle tracking echocardiography analysis of the indicators of cardiac deformation in relation to the postnatal diagnosis of small for gestational age (SGA).

	SGA *N* 16	Non SGA *N* 192	*p*
Maternal age, years Mean ± SD	30.3 ± 5.8	32.1 ± 5.3	0.19
Ethnicity *N* (%)	Caucasian 11 (68.8%) African 1 (6.2%) Asian 4 (25%) Other 0 (0%)	Caucasian 151 (78.6%) African 22 (11.5) Asian 8 (4.2%) Other 11 (5.7%)	< 0.01
Nulliparity *N* (%)	8 (50%)	129 (67.2%)	0.16
Previous caesarean delivery *N* (%)	2 (12.5%)	14 (7.3%)	0.45
Booking BMI, kg/m2 Mean ± SD	22.2 ± 2.8	23.8 ± 4.8	0.19
IVF conception *N* (%)	0 (0%)	12 (6.2%)	0.30
Smoking *N* (%)	2 (12.5%)	15 (7.8%)	0.51
GBS colonisation *N* (%)	3 (18.8%)	33 (17.2%)	0.36
Gestational age at recruitment, weeks^+days^ Mean ± SD	39^+4^ ± 1^+1^	39^+6^ ± 1^+1^	0.35
Baseline FHR at recruitment, bpm Mean ± SD	138 ± 11	141 ± 10	0.21
Number of contractions at recruitment Median (range)	4 (3–5)	4 (3–5)	0.82
Oxytocin administration *N* (%)	7 (43.8%)	130 (67.7%)	0.05
Epidural *N* (%)	11 (68.8%)	170 (88.5%)	0.02
Meconium stained amniotic fluid *N* (%)	3 (18.8%)	27 (14.1%)	0.61
Duration of ruptured membranes, minutes Mean ± SD	473 ± 448	810 ± 1092	0.24
First stage length, minutes Mean ± SD	202 ± 111	357 ± 209	< 0.01
Second stage length, minutes Mean ± SD	31 ± 32	59 ± 53	0.05
Mode of delivery *N* (%)	SVD 12 (75%) ID 2 (12.5%) CD 2 (12.5%)	SVD 156 (81.2%) ID 25 (13%) CD 11 (5.8%)	0.56
Operative delivery due to suspected IFC *N* (%)	3 (18.8%)	17 (8.9%)	0.20
Arterial pH Mean ± SD	7.16 ± 0.12	7.10 ± 0.13	0.10
Apgar at 5 min Median (range)	10 (2–10)	10 (6–10)	0.02
LV MyoGLS, % Mean ± SD	−16.3 ± 4.6	−15.5 ± 4.9	0.51
LV EndoGLS, % Mean ± SD	−18.6 ± 4.8	−17.4 ± 5.0	0.34
LV FAC, % Mean ± SD	31.9 ± 10.9	30.2 ± 9.1	0.47
LV EF, % Mean ± SD	44.5 ± 14.4	41.3 ± 12.4	0.33
RV MyoGLS, % Mean ± SD	−14.0 ± 5.3	−12.7 ± 4.3	0.24
RV EndoGLS, % Mean ± SD	−16.1 ± 5.5	−14.5 ± 4.7	0.19
RV FAC, % Mean ± SD	26.3 ± 8.6	24.9 ± 8.3	0.52

Abbreviations: BMI, body mass index; EF, ejection fraction; FAC, fractional area change; FHR, fetal heart rate; GBS, group B streptococcus; GLS, global longitudinal strain; GRS, global radial strain; IFC, intrapartum fetal compromise; IVF, in vitro fertilisation; LV, left ventricle; RV, right ventricle.

## 4 Discussion

### 4.1 Main Findings

In this exploratory study conducted on singleton term pregnancies which showed no sign of fetal hypoxia at enrolment, we demonstrate that myocardial deformation, particularly of the right ventricle, on admission in early labour is greater in cases eventually submitted to operative delivery due to suspected IFC. Of note, significant differences were noted only for the right ventricle, which in utero represents the ‘systemic’ ventricle and is exposed to the increased afterload determined by placental insufficiency. In cases undergoing emergency delivery due to intrauterine hypoxia, no differences in fetal myocardial deformational parameters at recruitment in early labour were noted related to the postnatal diagnosis of SGA birth.

### 4.2 Interpretation in Light of Current Evidence

The fetal heart plays a key role in the adaptation to intrauterine hypoxia [22]. Specifically, it has been demonstrated to undergo ‘a phase one’ subclinical adaptive changes in myocardial deformation aiming to sustain the stroke volume, hence the perfusion of the central organs, and preceding ‘a phase two’ abnormalities characterised by decreased cardiac output and stroke volume [23]. STE is acknowledged to represent a sensitive tool to assess the cardiac function and investigate the earliest hypoxic‐related adaptive changes in the fetus [10]. Studies evaluating fetal myocardial deformation are inconsistent and have mainly focused on fetal growth restriction (FGR) secondary to chronic placental insufficiency and hypoxia suggesting differences in terms of increased or reduced cardiac deformation between FGR and non‐growth restricted foetuses [24, 25, 26]. From a pathophysiology point of view, this can be explained by the fact that placental insufficiency determines an increase in the cardiac afterload for the right ventricle leading to cardiac adaptation to overcome the persistently increased umbilical artery resistance [24, 25, 26]. As such, a study investigating the fetal cardiac function prior to labour in apparently uncomplicated pregnancies with normally sized foetuses close to delivery showed lower cardiac deformation and ejection fraction in cases with apparently reduced placental functional reserve, that is, those eventually submitted to emergency operative delivery due to suspected IFC [27]. However, more recent evidence has shown that FGR can also be associated with cardiac remodelling of variable degree based on the extent and duration of the impaired placental function [28]. Of note, in postnatal studies conducted on infants born with FGR, such findings have been shown to persist into infancy and childhood [29, 30].

In this study we investigated the fetal heart response to intermittent and sustained intrauterine hypoxia characterising an early labour within a population at low risk of placental insufficiency. During uterine contractions the uteroplacental unit is under perfused determining the intermittent and sustained increase of the umbilical artery resistance and intrauterine hypoxia. These findings suggest that intermittent and sustained hypoxia in early labour may unmask preclinical fetal cardiac adaptation, potentially serving as an early marker for fetal compromise. In detail, an increased deformation of the right ventricular wall in the early stages of labour was found among the foetuses showing cardiotocographic features of intrapartum hypoxia at a later stage. We hypothesise that the adaptive response of the fetal heart may be more pronounced in cases with suboptimal placental function and whose tolerance to the ‘physiological’ hypoxic stress of labour is reduced. Such increased right ventricular deformation might be an anticipator of hypoxic intrapartum compromise and could be a result of the sympathetic drive caused by transient acute hypoxia occurring with the onset of labour. In other words, in some foetuses whose placental function is apparently normal at the onset of regular uterine contractions, the intermittent oxygen deprivation seems to unmask a pre‐existing condition of mild utero‐placental insufficiency [31] leading to subclinical adaptive changes of the fetal heart. Such changes aiming to sustain the cardiac output are mainly represented by an increased deformation of cardiac myocytes, which are mediated by neurohormonal factors such as catecholamines [32, 33] and precede clinical abnormalities which eventually become evident when labour progresses and fetal hypoxic stress defence becomes decompensated. Catecholamine release featuring labour has also been associated with the increased cardiac inotropism and fetal heart rate [34]. In such context, the higher baseline FHR noted in cases submitted to operative delivery due to suspected IFC is consistent with the hypothesis of sub‐optimally tolerated hypoxic stress with a resulting increased catecholamine release at the time of recruitment. Of note, the adaptive changes we demonstrate in this study become evident long before the appearance of overt signs of fetal hypoxia at CTG recording. Interestingly, subclinical adaptive changes in foetuses eventually delivered by operative delivery due to suspected IFC were noted only in the right ventricle. Such finding is not surprising as in intrauterine life the right ventricle is *de facto* the systemic ventricle exposed to the placental resistance due to the peculiar arrangement of the fetal circulation and to the presence of the arterial duct. Therefore, an increased umbilical artery resistance associated with uterine contractions impacts primarily on the function of the right ventricle, whilst left ventricular afterload is mostly influenced by the changes of resistance of the brain and heart vessels [35].

The findings of our study investigating subclinical functional changes of the fetal heart during active labour confirm that the reduced tolerance to the hypoxic stress of labour leading to operative delivery due to IFC is not dependent upon a fetal size. This supports the concept that placental insufficiency may remain subclinical before labour onset and be unmasked when uterine activity starts, irrespective of the fetal size at birth [31, 36, 37]. The fetal hemodynamic response to the hypoxic stress of labour in uncomplicated pregnancies has been investigated in previous works [38, 39, 40, 41]. Specifically, the assessment of fetal Doppler in early labour among low‐risk foetuses has been shown to improve to some extent the identification of the cases who eventually exhibit intrapartum fetal heart rate patterns indicating fetal hypoxia, albeit with limited accuracy and predictive value [38, 39, 40, 41]. However, compared with cardiac functional changes, Doppler anomalies are acknowledged to manifest only in the event of an advanced stage of fetal adaptation to intrauterine hypoxia [42]. The suboptimal predictive performance of Doppler ultrasound assessment in early labour for operative delivery due to IFC may also be interpreted in light of the concerns regarding the reproducibility of the Doppler measurements [19, 20, 43, 44, 45, 46].

### 4.3 Clinical and Research Implications

From a clinical point of view, our data supports the hypothesis that an increased fetal right ventricular longitudinal strain represents an early indicator of subclinical placental dysfunction which may be unmasked by the hypoxic stress elicited by uterine contractions. In our cohort, an increased RV strain was found in early labour among cases with normal CTG who were eventually submitted to operative delivery due to suspected IFC. In such context, STE analysis of the cardiac strain could be seen as a potential tool for the triage of the placental function and the associated risk of intrapartum hypoxia. However, more insights are required in terms of standardisation of data acquisition and analysis as uncertainty still exists as to whether the angle on insonation may impact on the output of the software [47, 48, 49]; furthermore, multicentre data are required to confirm the generalizability of the study findings, and reference ranges of normality vs. abnormality across gestation are yet to be defined. To date, none of the methods in use or proposed for the identification of the foetuses experiencing hypoxic events during labour, such as continuous CTG, intermittent auscultation and intrapartum Doppler has proved to be effective in improving perinatal outcomes [50, 51] or reducing interventions due to a false diagnosis of IFC.

In the present study, there was no correlation found between STE parameters and FHR obtained by CTG. In theory, the greater the hypoxic stress, the higher the release of catecholamines, and this is expected to increase the cardiac deformation but also the FHR baseline [52]. However, visual interpretation of the CTG allows only an estimation and not the accurate quantification of the actual baseline FHR as it can be computed using, for example, computerised CTG. Consistently, no relation was demonstrated between STE parameters and the actual number of contractions at the time of enrolment. Uterine activity may be considered the main trigger of the hypoxic stress in labour [53], and it may therefore be hypothesized that the higher the number of contractions, the greater the cardiac changes in myocardial deformation. However, on external tocography, only the frequency of contractions may be approximately estimated, while no accurate data on their strength and duration may be derived. On this basis, we cannot exclude that uterine activity in early labour, whether appropriately measured (i.e., intrauterine pressure catheter), is correlated with fetal cardiac strain responses.

### 4.4 Strengths and Limitations

To the best of our knowledge, this is the first study investigating the intrapartum fetal cardiac function using STE in relation to the labour outcome. The original and prospective design of the study as well as its rigorous methodology are the strengths of the research. The single centre design may limit the generalizability of the findings. Furthermore, the design of the study did not allow to evaluate potential confounders and sources of bias impacting on the results of the STE analysis of the myocardial strain such as the presence of pre‐existing cardiac remodelling. Another limitation is that the paired assessment of the fetal and maternal Doppler in early labour [38, 39, 40, 41] could have further supported the study findings and allowed to compare Doppler and STE analysis in terms of prediction of adverse outcome. Additionally, the estimated fetal weight was not evaluated on recruitment, which has precluded to compute z‐scores of the 4‐chamber width, global sphericity index, and area. However, it is worth observing that no difference in neonatal size was recorded between the cases having and those not having OD due to suspected IFC. Finally, we acknowledge that the primary outcome of our exploratory study—i.e. operative delivery due to suspected IFC—is subjective. More research is warranted to investigate the relationship between STE assessment of the fetal cardiac function and more objective labour outcomes (i.e., cord blood gas analysis).

Cardiac remodelling was also not investigated in this present study. While we included cases with no risk factor for in utero cardiac remodelling such as chronic hypoxia we acknowledge that such phenomena may also occur among foetuses without overt placental insufficiency. Indeed, the right afterload has been shown to longitudinally increase in the third trimester due to placental maturation and an elevated pulmonary vascular resistance prenatally [54, 55]. On this basis, even though our study aimed not to include cases featuring a chronic placental insufficiency, we cannot exclude that in some cases cardiac remodelling may have impacted the STE analysis of the cardiac deformation.

## 5 Conclusion

In singleton term pregnancies in early labour with normal CTG on admission, the myocardial deformation of the fetal heart, particularly of the right ventricle, is greater in foetuses submitted to operative delivery due to suspected IFC. The hypoxic stress of labour seems to trigger a compensatory response of the fetal heart to overcome the increased peripheral resistances and intrapartum hypoxia and sustain the cardiac output during labour.

## Author Contributions

Tullio Ghi: conceptualised the study, reviewed and revised the manuscript. Andrea Dall'Asta: conducted analyses, reviewed and revised the manuscript. Chiara Melito: led data collection and prepared the first draft. Beatrice Valentini, Mariagrazia Capurso and Maria Teresa Baffa: led data collection. Olga Patey and Basky Thilaganathan: reviewed and revised the manuscript. All authors critically reviewed the manuscript, including all revisions and agreed on the final version for submission to the journal.

## Ethics Statement

The study was approved by the Ethics Committee of the University Hospital of Parma [270/2018/OSS/AOUPR].

## Conflicts of Interest

The authors declare no conflicts of interest.

## Supporting information


**Data S1:** Table S1. Speckle tracking echocardiography analysis of the indicators of cardiac morphometry and function and sphericity index of the left and right ventricles in relation to the occurrence of obstetric operative delivery (OD) due to suspected intrapartum foetal compromise (IFC).

## Data Availability

Data are not publicly available due to ethical reasons but are available from the corresponding author (T.G.) upon reasonable request.
