# Supplementary material for: Fetal Cardiac Function in Early Labour and Intrapartum Outcomes: A Prospective Observational Study
Source: BJOG. 2025 May 21;133(7):1414–23. doi: 10.1111/1471-0528.18224 (PMC13143557; doi:10.1111/1471-0528.18224)
Supplement: Supplementary file 1 — Data S1: Table S1. Speckle tracking echocardiography analysis of the indicators of cardiac morphometry and function and sphericity index of the left and right ventricles in relation to the occurrence of obstetric operative delivery (OD) due to suspected intrapartum foetal compromise (IFC). [file BJO-133-1414-s001.docx]

Supplementary Table – Speckle tracking echocardiography analysis of the indicators of cardiac morphometry and function and sphericity index of the left and right ventricles in relation to the occurrence of obstetric operative delivery (OD) due to suspected intrapartum fetal compromise (IFC).

|  | ***OD due to suspected IFC***  ***N 20*** | ***Non OD due to suspected IFC***  ***N 188*** | ***p*** |
| --- | --- | --- | --- |
| **LV EDA**  **Mean + SD** | 5.7 + 4.4 | 6.8 + 6.2 | 0.45 |
| **LV ESA**  **Mean + SD** | 3.8 + 3.0 | 4.8 + 4.5 | 0.36 |
| **LV ESL**  **Mean + SD** | 2.8 + 0.9 | 3.0 + 1.2 | 0.48 |
| **LV ESD bas**  **Mean + SD** | 1.7 + 0.7 | 1.7 + 0.8 | 0.63 |
| **LV ESD mid**  **Mean + SD** | 1.4 + 0.6 | 1.5 + 0.7 | 0.41 |
| **LV EDL**  **Mean + SD** | 3.4 + 1.1 | 3.7 + 1.4 | 0.39 |
| **LV EDD bas**  **Mean + SD** | 1.7 + 0.8 | 1.8 + 0.9 | 0.57 |
| **LV EDD mid**  **Mean + SD** | 1.7 + 0.7 | 1.8 + 0.8 | 0.53 |
| **LV EDV**  **Mean + SD** | 9.5 + 13.3 | 13.0 + 20.2 | 0.45 |
| **LV ESV**  **Mean + SD** | 5.3 + 6.9 | 7.9 + 12.7 | 0.37 |
| **iMAPSE**  **Mean + SD** | 0.7 + 0.3 | 0.7 + 0.4 | 0.89 |
| **sMAPSE**  **Mean + SD** | 0.5 + 0.2 | 0.6 + 0.3 | 0.43 |
| **LV MyoArea**  **Mean + SD** | 2.8 + 2.2 | 3.7 + 3.9 | 0.31 |
| **LV sphericity index**  **Mean + SD** | 1.6 + 0.3 | 1.9 + 0.4 | 0.30 |
| **RV EDA**  **Mean + SD** | 6.5 + 4.8 | 7.2 + 6.4 | 0.60 |
| **RV ESA**  **Mean + SD** | 4.9 + 3.9 | 5.5 + 5.1 | 0.63 |
| **RV ESL**  **Mean + SD** | 2.7 + 0.8 | 3.0 + 1.2 | 0.32 |
| **RV ESD bas**  **Mean + SD** | 1.9 + 0.7 | 1.9 + 0.8 | 0.92 |
| **RV ESD mid**  **Mean + SD** | 1.8 + 0.8 | 1.8 + 0.8 | 0.89 |
| **RV EDL**  **Mean + SD** | 3.2 + 0.9 | 3.5 + 1.3 | 0.34 |
| **RV EDD bas**  **Mean + SD** | 2.0 + 0.7 | 2.0 + 0.9 | 0.93 |
| **RV EDD mid**  **Mean + SD** | 2.1 + 0.8 | 2.1 + 0.8 | 0.97 |
| **RV Free Wall ST**  **Mean + SD** | -17.7 + 5.3 | -15.3 + 6.6 | 0.12 |
| **TAPSE Mean + SD** | 0.6 + 0.2 | 0.5 + 0.3 | 0.45 |
| **RV MyoArea**  **Mean + SD** | 3.1 + 2.1 | 3.6 + 3.9 | 0.58 |
| **RV sphericity index**  **Mean + SD** | 1.7 + 0.4 | 1.6 + 0.3 | 0.10 |
